# A critical role of hippocampus for formation of remote cued fear memory

**DOI:** 10.1186/s13041-020-00652-y

**Published:** 2020-08-15

**Authors:** Jung-Pyo Oh, Jin-Hee Han

**Affiliations:** 1 Department of Biological Sciences, Korea Advanced Institute of Science and Technology (KAIST), 291 Daehak-ro, Yuseong-gu, Daejeon, 34141 Republic of Korea; 2 KAIST Institute for the BioCentury (KIB), Korea Advanced Institute of Science and Technology (KAIST), 291 Daehak-ro, Yuseong-gu, Daejeon, 34141 Republic of Korea

**Keywords:** Hippocampus, Reactivation, Auditory fear conditioning, Remote memory

## Abstract

A unique feature of fear memory is its persistence that is highly relevant to fear and anxiety-related mental disorders. Recurrent reactivation of neural representations acquired from a traumatic event is thought to contribute to the indelibility of fear memory. Given a well-established role of hippocampus for memory reactivation, hippocampus is likely involved in consolidation process of fear memory. However, evidence suggests that formation of fear memory to a discrete sensory cue is hippocampus-independent. Here, using a pharmacological reversible inactivation of dorsal hippocampus in auditory cued fear conditioning by local infusion of muscimol, we demonstrate in mice that hippocampus is critical for remote memory formation of learned fear to the discrete sensory cue. Muscimol infusion before conditioning did not affect formation of recent auditory fear memory as previously reported. Same muscimol infusion, however, impaired remote auditory fear memory. Muscimol infusion before remote test of auditory fear memory did not affect memory retrieval, indicating hippocampus is not a brain site for storage of remote cued fear memory. Moreover, memory reactivation enforced by re-exposure to the conditioned tone could compensate for hippocampal inactivation, as memory-reactivated mice showed normal remote auditory fear memory despite hippocampal inactivation. Our findings support that hippocampus may have a general role for consolidation of remote associative memory through reactivation of memory trace, giving an insight into how learned fear persists over time.

## Introduction

Memory about whether any particular sensory stimulus predicts danger is essential for survival of animals. This so-called fear memory is unique among many different types of memory. It is readily formed in the brain when experiencing traumatic events and remains intact for a whole lifetime of the organism without forgetting [1, 2]. This endurance represents a highly adaptive function of fear memory and a major cause of maladaptive fear and anxiety-related mental disorders. In the laboratory, Pavlovian fear conditioning has been used as an experimental model to study associative fear learning and memory in animals. In Pavlovian fear conditioning, an emotionally neutral sensory stimulus (conditioned stimulus, CS) such as light or tone is paired with an aversive stimulus (unconditioned stimulus, US) such as a footshock. As a consequence of conditioning, CS comes to elicit fear-related defensive responses such as freezing, an index of fear memory.

Studies using fear conditioning paradigm in rodents identified a widely distributed interconnected neuronal network underlying the acquisition and expression of learned fear including the amygdala, medial prefrontal cortex (mPFC), sensory cortices and hippocampus [3, 4]. It is generally believed that the amygdala, especially basolateral complex (BLA), is a core brain locus for encoding and permanent storage of learned fear [2, 4–8]. In contrast, hippocampus is thought to be selectively involved in the formation and consolidation of fear memory to context with no substantial contribution to discrete cued fear memory formation [5, 9]. Only under certain learning conditions, hippocampus may contribute to the cued fear memory formation [10–14]. Consistent with the specific involvement of hippocampus in contextual fear memory, pre-training infusion of muscimol, a GABA_A_ receptor agonist, into dorsal hippocampus (DH, hereafter) impairs contextual fear memory formation but no effect on cued fear memory formation [13, 15, 16]. Moreover, post-learning reversible inactivation of DH by tetrodotoxin (TTX) selectively disrupts contextual but not cued fear memory in rats [17]. Despite these findings, it is still unclear whether hippocampus has any role for persistence of fear memory because most prior studies have focused on recent memory formation. A recent study in rats shows hippocampus is selectively crucial for remote, but not recent, memory formation in a novel object recognition task, which is also known as hippocampus-independent behavior paradigm [18]. So, in this study, we examined whether hippocampus is involved in remote memory formation of conditioned fear by using a pharmacological inactivation of DH and auditory fear conditioning paradigm in mice.

## Results

### Pre-training muscimol infusion in the DH impairs recent contextual, but not auditory cued fear memory

**Fig. 1:**
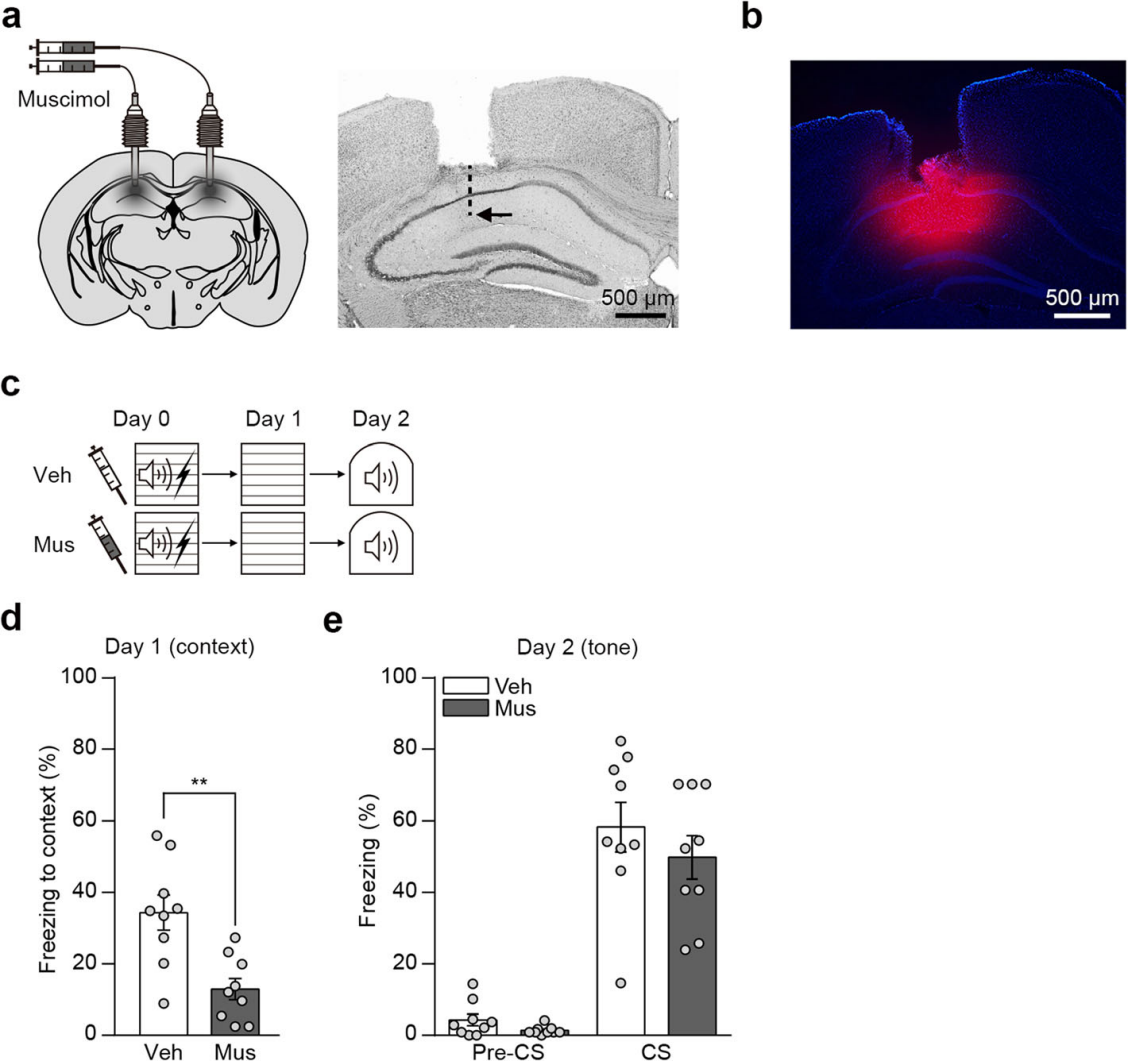
Pre-training muscimol infusion in the DH impairs recent contextual, but not auditory cued fear memory. **a** Left, Cannula implantation and drug infusion strategy in DH. Right, Representative brain section showing cannula placement above DH. Arrow indicates estimated location of injection cannula. **b** Representative brain section showing the spread of muscimol. TMR-X conjugated muscimol was infused to estimate the infusion range of muscimol. **c** Behavior procedures. **d** Histogram showing freezing levels to conditioned context (*n* = 9 per group; two-tailed Student’s *t*-test, *t*_(16)_ = 3.696, **p < 0.01). **e** Histogram showing freezing levels to conditioned tone CS (*n* = 9 per group; two-way repeated-measures ANOVA followed by Bonferroni’s *post-hoc* test). Data are mean ± s.e.m. DH, dorsal hippocampus

For hippocampus inactivation during auditory cued fear conditioning, we administered muscimol bilaterally into the DH through cannulas 15 min before the training (Fig. 1a). Using TMR-X conjugated muscimol, we confirmed targeted infusion of drug in DH (Fig. 1b). We first examined whether previous reports on recent memory formation are reproduced in our condition. Two groups of mice infused either with vehicle or muscimol were trained for auditory fear conditioning and next day tested for contextual fear memory. One day later the same mice were tested for cued fear memory to the tone CS (Fig. 1c). Consistent with previous reports, we found contextual fear memory is selectively impaired in this condition. Mice in muscimol group displayed a significantly lower level of context freezing compared to mice in vehicle control group (Fig. 1d). In contrast, no significant difference was observed in tone freezing between groups (Fig. 1e). These results confirm that DH is not essential for recent cued fear memory formation.

### Inactivation of the DH impairs remote auditory fear memory formation but not retrieval

**Fig. 2:**
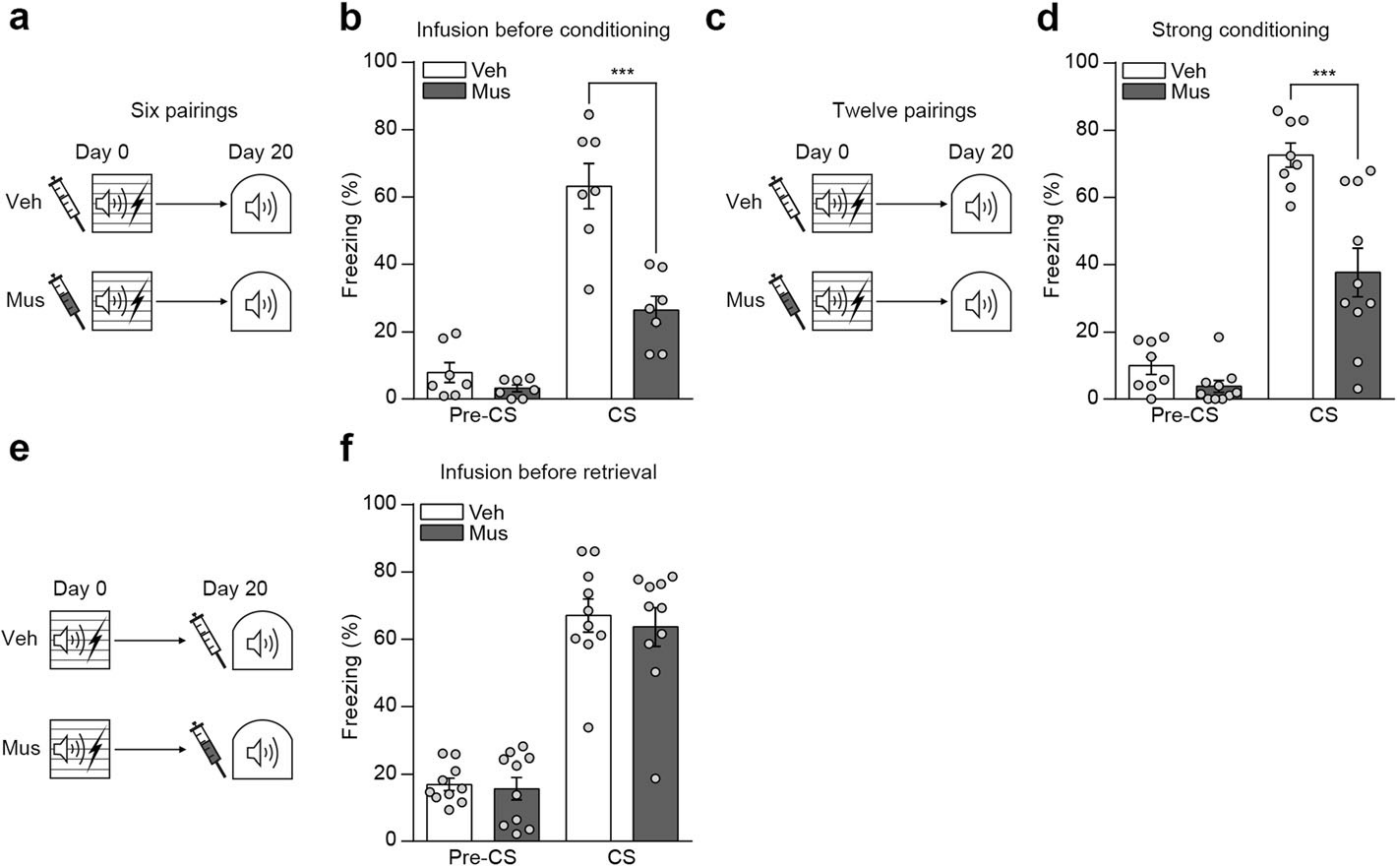
Inactivation of the DH impairs remote auditory fear memory formation but not retrieval. **a** Behavior procedures. **b** Histogram showing freezing levels to conditioned tone (*n* = 7 per group; two-way repeated-measures ANOVA followed by Bonferroni’s *post-hoc* test, ***p < 0.001). **c** Behavior procedures. **d** Histogram showing freezing levels to conditioned tone (vehicle, *n* = 8; muscimol, *n* = 10; two-way repeated-measures ANOVA followed by Bonferroni’s *post-hoc* test, ***p < 0.001). **e** Behavior procedures. **f** Histogram showing freezing levels to conditioned tone (*n* = 10 per group; two-way repeated-measures ANOVA followed by Bonferroni’s *post-hoc* test). Data are mean ± s.e.m.

Next, we examined the effect of hippocampus inactivation by muscimol infusion on remote cued fear memory. We used same procedures as above except in this experiment mice were tested twenty days after the training (Fig. 2a). Different from the recent memory test, we found that remote memory was severely impaired in mice received hippocampus inactivation. Mice in muscimol group displayed significantly less conditioned freezing to the tone CS compared to mice in control group (Fig. 2b). The memory impairment at remote, not recent, time may simply reflect a time-dependent decay of memory because association strength was not strong enough in this learning condition (6 trials of tone-shock pairing). To test this possibility, we delivered 12 trials of tone-shock pairing to mice during training (Fig. 2c). Muscimol or vehicle as a control was administered as before. In this experimental condition, we again observed fear memory impairment at remote time in muscimol group. When mice were tested 20 days after training, mice in muscimol group displayed significantly less freezing to the tone compared to mice in vehicle control group (Fig. 2d). This result therefore further supports that DH function is specifically required for forming remote auditory fear memory. One possible account for this remote specific memory impairment is that hippocampus may be gradually involved in representations of cued fear memory over time and thus required for retrieval of remote cued fear memory. If this is the case, hippocampus inactivation during remote memory test should disrupt memory retrieval. To examine this possibility, muscimol was administered 15 min before retrieval test of remote memory instead of before training (Fig. 2e). We found no significant effect in this condition. Mice infused with muscimol displayed normal conditioned freezing to the tone comparable to that in control mice (Fig. 2f). So, it is unlikely that DH is involved in storage or retrieval of remote cued fear memory. Together, these results suggest that hippocampus is crucial for consolidation of remote cued fear memory.

### Reactivation of memory by re-exposure to the conditioned tone rescues remote memory impairment by DH inactivation

**Fig. 3:**
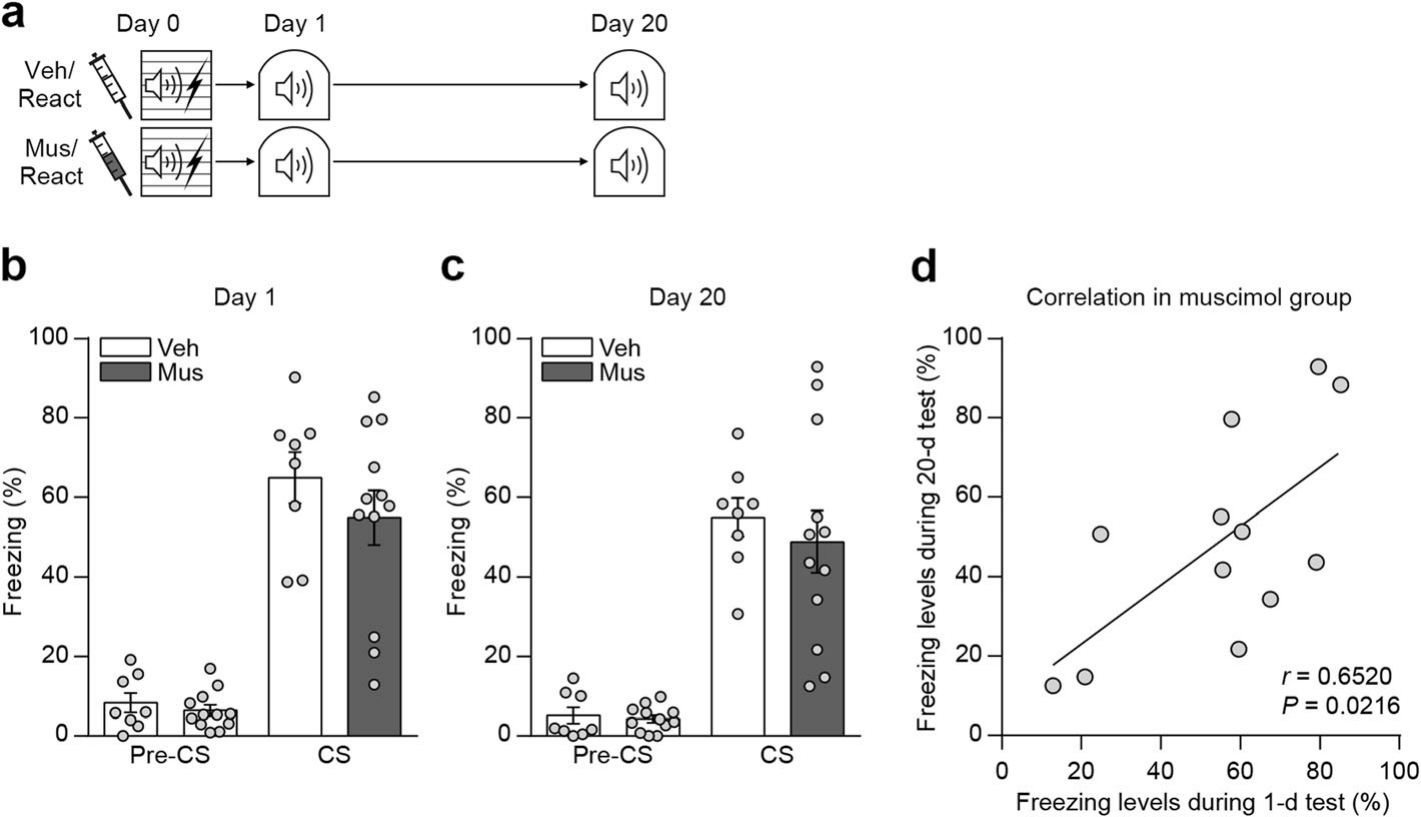
Reactivation of memory by re-exposure to the conditioned tone compensates for DH inactivation. **a** Behavior procedures. **b** Histogram showing freezing levels to conditioned tone during recent test (vehicle, *n* = 8; muscimol, *n* = 12; two-way repeated-measures ANOVA followed by Bonferroni’s *post-hoc* test). **c** Histogram showing freezing levels to conditioned tone during remote test (vehicle, *n* = 8; muscimol, *n* = 12; two-way repeated-measures ANOVA followed by Bonferroni’s *post-hoc* test). **d** Correlation analysis using data from muscimol group (*n* = 12, *r* = 0.6520, *p* = 0.0216, Pearson correlation coefficient). Data are mean ± s.e.m.

Hippocampus is thought to consolidate memories by promoting internal reactivation of neuronal activity patterns present during learning events [19]. We reasoned that remote memory deficit of cued fear conditioning formed without intact DH may be related with such reactivation function of hippocampus. To examine this idea, we asked whether reactivating the memory by re-exposing to the conditioned sensory cue can compensate for hippocampus inactivation. As before, mice received the local infusion of either muscimol or vehicle into the bilateral DH and 15 min later underwent auditory fear conditioning. For the purpose of memory reactivation, next day mice were re-exposed to the conditioned tone in a novel context just as recent memory test and tested again for the remote memory retention 19 days later (Fig. 3a). During the reactivation session, we again confirmed DH is not essential for recent cued fear memory formation (Fig. 3b). In this reactivation experimental condition, however, we found that mice trained with inactivation of DH displayed normal remote cued fear memory compared to control mice (Fig. 3c), consistent with the compensation by cued-dependent reactivation. Correlation analysis revealed a significant positive correlation in freezing levels between recent and remote memory test in each individual animal from muscimol group, suggesting the memory retention by reactivation (Fig. 3d).

### Re-exposure to the conditioned tone but not a novel context is responsible for rescue of remote memory impairment

**Fig. 4:**
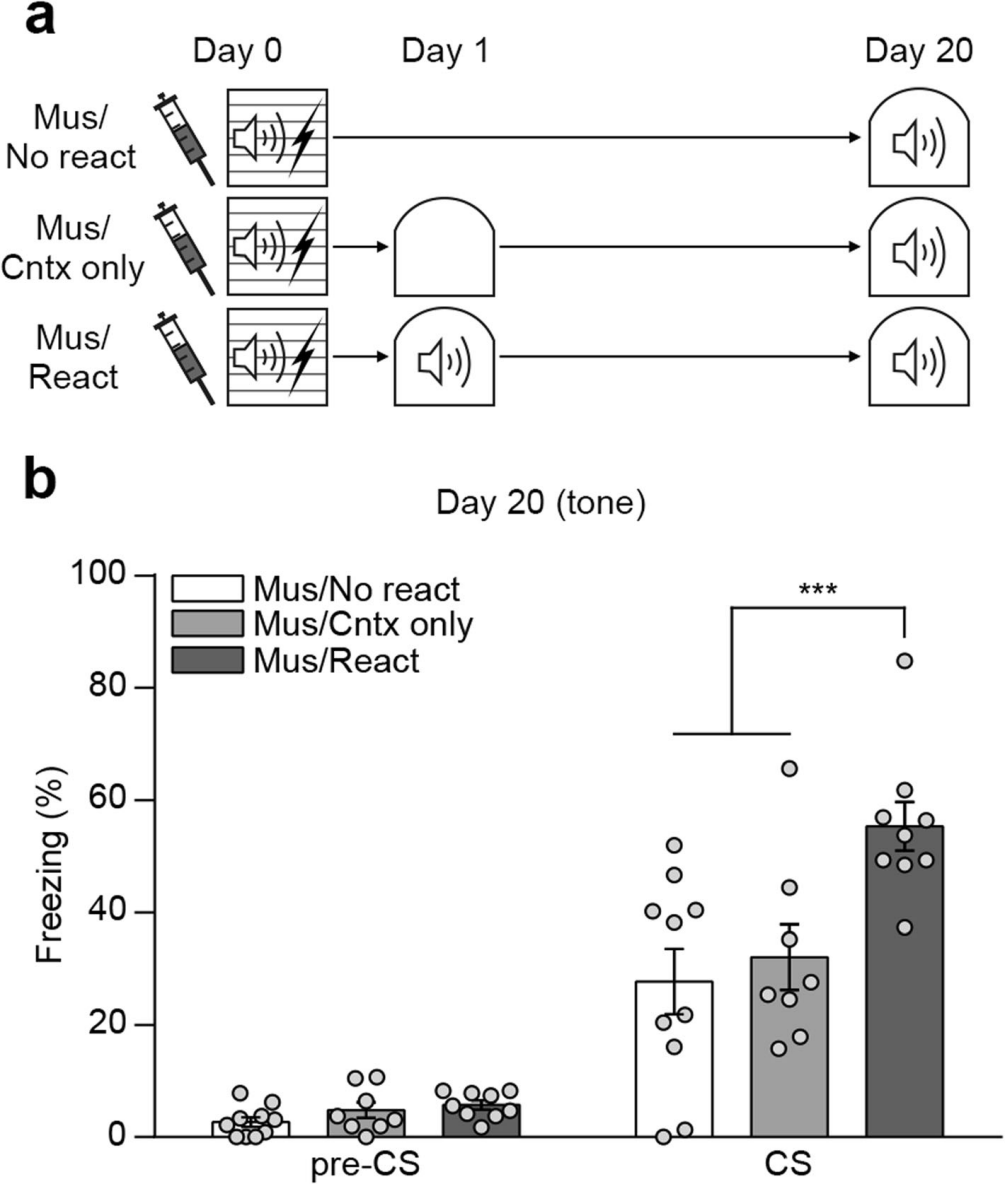
Re-exposure to the conditioned tone is essential to rescue remote memory impairment. **a** Behavior procedures. **b** Histogram showing freezing levels to conditioned tone during remote test (Mus/No react, *n* = 10; Mus/Cntx only, *n* = 8; Mus/React, *n* = 9; two-way repeated-measures ANOVA followed by Bonferroni’s *post-hoc* test, *** *p* < 0.001). Data are mean ± s.e.m.

To further confirm the reactivation effect, we performed a specificity test. During the reactivation session, mice are exposed to not only the conditioned tone but also a novel context in a test chamber. Thus, we examined whether exposure to the context alone can elicit a similar effect on remote memory retention with tone exposure condition. All three groups of mice received local infusion of muscimol and underwent auditory fear conditioning as before. The next day, reactivation group was exposed to the conditioned tone in a novel context as before whereas context only group was placed in the same novel context but without tone. Homecage control group kept remained in their homecage after learning until retrieval test. All mice were tested for remote memory retention 20 days after fear conditioning (Fig. 4a). During the remote test, mice in context only and homecage control group exhibited significantly lower level of freezing to the conditioned tone compared to mice in reactivation group. There was no significant difference in the freezing levels between control groups (Fig. 4b). These results confirm that memory reactivation by re-exposure to the conditioned tone rescued remote memory impairment in DH inactivated mice.

### Re-exposure to the conditioned context does not rescue remote memory impairment

**Fig. 5:**
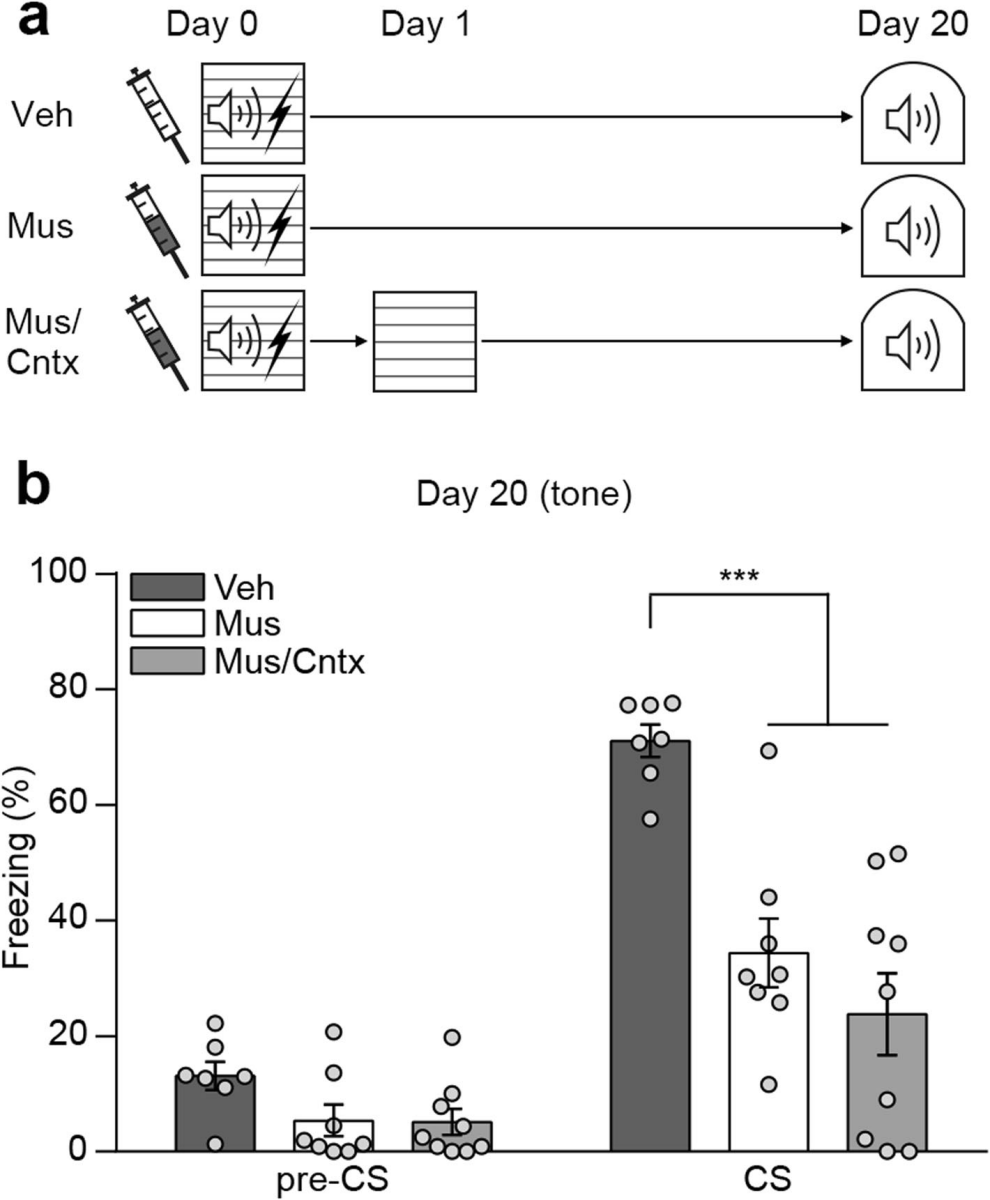
Re-exposure to the conditioned context does not rescue remote memory impairment. **a** Behavior procedures. **b** Histogram showing freezing levels to conditioned tone during remote test (Veh, *n* = 7; Mus, *n* = 8; Mus/Cntx, *n* = 9; two-way repeated-measures ANOVA followed by Bonferroni’s *post-hoc* test, *** *p* < 0.001). Data are mean ± s.e.m.

DH inactivation by muscimol infusion is thought to be transient in our condition [16, 20], meaning that DH function was intact when mice were re-exposed to the conditioned tone in the above experiments. This raises a possibility that reactivation of fear memory by re-exposure to the conditioned tone was still mediated by DH function for the rescue of remote memory impairment. To address this question, we next examined whether re-exposure to the conditioned context without tone CS could rescue remote memory impairment in mice trained with DH inactivation. As before, mice were administered with either muscimol or vehicle and trained for auditory fear conditioning. 1 day after training, one group of muscimol infused mice re-entered to the same chamber where associative learning events occurred during training (Mus/Cntx group), while the other group remained in their homecage (Mus group). All mice were tested for remote memory retention 20 days after conditioning (Fig. 5a). During the remote memory test, mice from both Mus/Cntx and Mus displayed a significantly less freezing to the conditioned tone compared to vehicle control. There was no significant difference in freezing levels between Mus/Cntx and Mus group (Fig. 5b). These results indicate that re-exposure to the conditioned context does not rescue the impairment of remote auditory fear memory formation and so it is unlikely that the rescue effect of re-exposure to the conditioned tone was mediated by DH function.

## Discussion

Our results here identify the crucial role of the hippocampus for remote memory formation of cued fear conditioning. Consistent with numerous prior reports, we also confirmed in this study that DH is not essential for recent cued fear memory formation. No effect of hippocampus inactivation on the retrieval of remote memory demonstrates that hippocampus is not crucial for retrieval and not a storage site of remote cued fear memory. These results together suggest that hippocampus is involved in consolidation process of remote cued fear memory. Previously, it was suggested in rats that DH is involved in cued fear conditioning under relatively weak associative learning conditions [14]. However, the recent memory formation was normal in our condition and the level of conditioned freezing is comparable to that of strong association conditions from Quinn et al. 2008. Moreover, we observed the same impairment of remote auditory fear memory even in stronger conditioning condition. Given these findings, it is unlikely that our results reflect the hippocampus function related with weak associative learning conditions.

Because we used a drug muscimol for inactivation of DH, our manipulation has limitations of spatial and temporal specificity. To estimate the diffusion range of muscimol, we infused TMR-X conjugated muscimol with the same condition used in behavior experiments [16, 18]. Although we observed a majority of fluorescent signals in the dorsal part of hippocampus with no signal detected in the ventral part, there were also some fluorescence signals in the sensorimotor cortex around the cannula tract likely due to spillover of injection solution through the cannula. Thus, it is possible that fear memory impairment in muscimol groups resulted from somatosensory cortex inactivation by the unintended spread of muscimol. However, we think that this is unlikely based on the following reasons. First, such cortical region was also damaged by cannula implantation even in the vehicle control groups, but fear memory formation was normal in those control animals. Second, previous findings showed that lesions made in the same cortical regions located above DH do not affect fear conditioning to both context and discrete auditory cue [5, 9], demonstrating such cortical area is not necessary for fear memory formation.

It is unknown in this study how hippocampus contributes to the formation of remote cued fear memory. Considering the well-known role of hippocampus for memory consolidation through reactivation activity, our data support the idea that representation of episodic events of cued fear conditioning may be formed in the hippocampus at the time of learning and drive an internal reactivation of cells in the extra-hippocampal brain areas that needs for the time-dependent reconstruction of a memory across broad brain networks for the permanent storage [4, 21–26]. According to this view, intact hippocampal system is required at the time of learning for subsequent remote memory consolidation. Supporting the idea of hippocampal driven reactivation of cued fear memory, previous studies show a positive correlation between theta-phase synchronization from hippocampal CA1 to amygdala during post-learning sleep and subsequent fear retrieval [27, 28]. Our finding that memory reactivation enforced by re-exposure to the conditioned sensory cue could rescue remote memory impairment in mice trained without intact hippocampus also supports this possibility. Re-exposure to the conditioned context had no rescue effect on remote memory. Our interpretation of this result is that the context-related representations that can induce reactivation of auditory fear memory representation in the extra-hippocampal brain areas that is needed during consolidation for the permanent storage of auditory cued fear memory was not formed in the hippocampus of muscimol infused mice due to inactivation of DH during conditioning. Therefore, even though mice were re-exposed to the conditioned context, there was no reactivation effect on memory.

Although we here focused on auditory fear memory, similar mechanism may underlie systems consolidation of hippocampus-dependent memories such as contextual fear memory. Indeed, activity of cortical regions such as anterior cingulate cortex (ACC) that are involved in remote storage of hippocampus-dependent memories during systems consolidation is shown to affect remote memory formation [29–33]. Such cortical activity during consolidation may be driven by activation of context-related representations formed in the hippocampus [34, 35]. Several brain structures have been suggested as a remote memory storage site for cued associative fear memory such as a higher order sensory cortex [21, 36], auditory thalamus [36], retrosplenial cortex [25], and paraventricular nucleus of thalamus [24]. Interestingly, pharmacological inactivation of temporal association cortex Te2 with TTX or muscimol 1 day following auditory fear conditioning impairs remote memory formation [23]. How memory reactivation by re-exposure to the external sensory cue keeps remote memory intact is an intriguing question that needs to be addressed in the future study.

Our results here support the perspective that hippocampus plays a critical role for consolidation of associative fear memory and give insights into how to improve memory in brain diseases with hippocampal damage.

## Materials and methods

### Mice

129S6/SvEvTac × C57BL/6 J hybrid male mice (2–3 months old, 23–35 g, overall 141 males) were group-housed on a 12-h light/dark cycle at a constant temperature of 22 ± 1 °C with 40–60% humidity. Mice were single housed after implantation of guide cannula. Food and water were available ad libitum throughout the experiment. All procedures were approved by the Animal Ethics Committee at the Korea Advanced Institute of Science and Technology (KAIST).

### Surgery

Mice were anesthetized by intraperitoneal injection of pentobarbital (83 mg per kg) and fixed in a stereotaxic frame. For muscimol infusion in dorsal hippocampus, guide cannula (2-mm guide, Plastics One, C313GS-5) were implanted right above bilateral dorsal hippocampus and fixed with dental cement. Coordinate relative to Bregma was AP − 1.9 mm, ML ±1.7 mm, DV − 1.0 mm. The injection cannula protruded 0.5 mm below guide cannula. Mice were given at least 7 d for recovery before the behavior experiments.

### Fear conditioning

After 7-d recovery from guide cannula implantation, mice were handled and habituated with injection cannula. For hippocampal inactivation during auditory fear conditioning, drug was infused 15 min before training. For auditory fear conditioning, mice were placed in fear conditioning chamber (Coulbourn Instruments) with video camera monitoring. Two minutes later, mice received six pairings of the pure tone CS (2.8 kHz, 85 dB, 20-s duration) followed by co-terminating US (0.4-mA shock, 2-s duration) with random inter-trial interval given at an average of 2 min. For strong conditioning, mice received twelve pairings of tone CS and US. Mice were left in the chamber for an additional 30 s after delivery of the last shock, and then moved back to their home cage.

For the contextual fear memory test, mice were placed in a conditioned context 1 day after conditioning. Mice were left in conditioned context for 3 min and moved back to their home cage. The freezing behavior during 3-min context exposure were measured as an index for contextual fear memory.

For hippocampal inactivation during auditory fear retention at remote time point, drug was infused 15 min before retention. For the auditory fear memory test, mice were placed in a context-shifted test chamber at a day indicated in each figure panel. After the 2-min baseline recording, 1-min tone CS was presented and mice were removed from the test chamber after an additional 30 s following termination of tone. For Mus/Cntx only group in Fig. 4, mice were place in a context-shifted test chamber for 3.5 min without tone presentation. For Mus/Cntx group in Fig. 5, mice were placed in a conditioned context for 3 min. The freezing behavior for first 2 min before tone presentation and 1 min during tone presentation were measured as index of baseline and tone CS-induced fear memory. Freezing behavior was automatically scored using FreezeFrame software (Actimetrics).

### Drug infusion

Muscimol (Sigma, M1523), GABA_A_ receptor agonist, was locally infused to dorsal hippocampus to block hippocampal activity during auditory fear conditioning or retrieval. To estimate the infusion range of muscimol, TMR-X conjugated muscimol (Molecular probes, M23400) was used, and the mice were perfused immediately after infusion of TMR-X conjugated muscimol. Muscimol was diluted in filtered PBS solution as a concentration of 2 mg per ml. For infusion of muscimol or vehicle PBS, injection cannula connected to 10-μl Hamilton syringe were filled with drug solution and inserted into guide cannula. Total 0.25 μl (0.5 μg of muscimol) of solution was infused into each hemisphere. Infusion rate was 0.2 μl per min. Injection cannula were left in place for additional 3 min to allow diffusion and removed. Behavior experiments were conducted 15 min after administration of drug.

### Histology

Mice were transcardially perfused with PBS, followed by 4% paraformaldehyde (PFA) after behavior test. Extracted brains were incubated in 4% PFA solution for overnight at 4 °C. Coronal brain sections (40-μm thickness) were obtained using Vibratome (Leica, VT1200S). For verification of the guide cannula placement, sections were counterstained with 1% Neutral red (Sigma, N4638) and mounted with xylene-containing Cytoseal (Thermo Scientific, #8311). Sections were observed under a bright-field view of Nikon Eclipse microscope (Nikon, 80i). The location of injection cannula tips for mice included in the analysis are presented in Fig. S1, S2.

For sections used to estimate the spread of TMR-X conjugated muscimol, sections were mounted with Vectashield media with DAPI (H-1200-10, Vector Laboratories) and observed under Nikon Eclipse microscope.

### Statistical analysis

All statistical analyses were performed using GraphPad Prism Version 6.07. Statistical details, including statistical tests used and exact values of *n*, are found in the figure/figure legends. Data are presented as mean ± s.e.m. Statistical significance was determined using Student’s *t*-test (two-tailed), two-way repeated-measures analysis of variance (ANOVA), followed by Bonferroni post hoc test for multiple comparisons with a significance level of α = 0.05 (n.s. *p* > 0.05, *p < 0.05, **p < 0.01, ***p < 0.001). To analyze the correlation in freezing levels between 1-d and 20-d test, Pearson correlation coefficient was calculated. No statistical methods were used to predetermine sample sizes, and sample sizes were determined based on previous studies.

## Supplementary information

**Additional file 1: Figure S1.** Schematic illustrations of injection cannula placements in mice used for behavior experiments. Related to Figs. 1, 2.

**Additional file 2: Figure S2.** Schematic illustrations of injection cannula placements in mice used for behavior experiments. Related to Fig. 3, 4 and 5.

## Data Availability

The datasets generated and analyzed during the current study are available from the corresponding author upon reasonable request.

## Abbreviations

DH: Dorsal hippocampus
mPFC: Medial prefrontal cortex
BLA: Basolateral complex of amygdala
CS: Conditioned stimulus
US: Unconditioned stimulus
TTX: Tetrodotoxin
PBS: Phosphate-buffered saline
PFA: Paraformaldehyde

## References

[1] Fanselow MS (1990). Factors governing one-trial contextual conditioning. Anim Learn Behav.

[2] Gale GD, Anagnostaras SG, Godsil BP, Mitchell S, Nozawa T, Sage JR (2004). Role of the basolateral amygdala in the storage of fear memories across the adult lifetime of rats. J Neurosci.

[3] Tovote P, Fadok JP, Lüthi A (2015). Neuronal circuits for fear and anxiety. Nat Rev Neurosci.

[4] Bergstrom HC (2016). The neurocircuitry of remote cued fear memory. Neurosci Biobehav Rev.

[5] Phillips R, LeDoux J (1992). Differential contribution of amygdala and hippocampus to cued and contextual fear conditioning. Behav Neurosci.

[6] Maren S, Aharonov G, Fanselow MS (1996). Retrograde abolition of conditional fear after excitotoxic lesions in the basolateral amygdala of rats: absence of a temporal gradient. Behav Neurosci.

[7] Fanselow MS, LeDoux JE (1999). Why we think plasticity underlying Pavlovian fear conditioning occurs in the basolateral amygdala. Neuron..

[8] Poulos AM, Li V, Sterlace SS, Tokushige F, Ponnusamy R, Fanselow MS (2009). Persistence of fear memory across time requires the basolateral amygdala complex. Proc Natl Acad Sci U S A.

[9] Kim JJ, Fanselow MS (1992). Modality-specific retrograde amnesia of fear. Science..

[10] Richmond M, Yee B, Pouzet B, Veenman L, Rawlins J, Feldon J (1999). Dissociating context and space within the hippocampus: effects of complete, dorsal, and ventral excitotoxic hippocampal lesions on conditioned freezing and spatial learning. Behav Neurosci.

[11] Zhang WN, Bast T, Feldon J (2001). The ventral hippocampus and fear conditioning in rats: different anterograde amnesias of fear after infusion of N-methyl-D-aspartate or its noncompetitive antagonist MK-801 into the ventral hippocampus. Behav Brain Res.

[12] Bast T, Zhang WN, Feldon J (2003). Dorsal hippocampus and classical fear conditioning to tone and context in rats: effects of local NMDA-receptor blockade and stimulation. Hippocampus..

[13] Maren S, Holt WG (2004). Hippocampus and Pavlovian fear conditioning in rats: muscimol infusions into the ventral, but not dorsal, hippocampus impair the acquisition of conditional freezing to an auditory conditional stimulus. Behav Neurosci.

[14] Quinn JJ, Wied HM, Ma QD, Tinsley MR, Fanselow MS (2008). Dorsal hippocampus involvement in delay fear conditioning depends upon the strength of the tone-footshock association. Hippocampus..

[15] Raybuck JD, Lattal KM (2011). Double dissociation of amygdala and hippocampal contributions to trace and delay fear conditioning. PLoS One.

[16] Misane I, Kruis A, Pieneman AW, Ögren SO, Stiedl O (2013). GABA_A_ receptor activation in the CA1 area of the dorsal hippocampus impairs consolidation of conditioned contextual fear in C57BL/6J mice. Behav Brain Res.

[17] Sacchetti B, Lorenzini CA, Baldi E, Tassoni G, Bucherelli C (1999). Auditory thalamus, dorsal hippocampus, basolateral amygdala, and perirhinal cortex role in the consolidation of conditioned freezing to context and to acoustic conditioned stimulus in the rat. J Neurosci.

[18] Sawangjit A, Oyanedel CN, Niethard N, Salazar C, Born J, Inostroza M (2018). The hippocampus is crucial for forming non-hippocampal long-term memory during sleep. Nature..

[19] Rasch B, Born J (2013). About sleep's role in memory. Physiol Rev.

[20] Corcoran KA, Desmond TJ, Frey KA, Maren S (2005). Hippocampal inactivation disrupts the acquisition and contextual encoding of fear extinction. J Neurosci.

[21] Sacco T, Sacchetti B (2010). Role of secondary sensory cortices in emotional memory storage and retrieval in rats. Science..

[22] Grosso A, Cambiaghi M, Concina G, Sacco T, Sacchetti B (2015). Auditory cortex involvement in emotional learning and memory. Neuroscience..

[23] Grosso A, Cambiaghi M, Renna A, Milano L, Merlo GR, Sacco T (2015). The higher order auditory cortex is involved in the assignment of affective value to sensory stimuli. Nat Commun.

[24] Do-Monte FH, Quinones-Laracuente K, Quirk GJ (2015). A temporal shift in the circuits mediating retrieval of fear memory. Nature..

[25] Todd TP, Mehlman ML, Keene CS, DeAngeli NE, Bucci DJ (2016). Retrosplenial cortex is required for the retrieval of remote memory for auditory cues. Learn Mem.

[26] Alvarez P, Squire LR (1994). Memory consolidation and the medial temporal lobe: a simple network model. Proc Natl Acad Sci U S A.

[27] Popa D, Duvarci S, Popescu AT, Léna C, Paré D (2010). Coherent amygdalocortical theta promotes fear memory consolidation during paradoxical sleep. Proc Natl Acad Sci U S A.

[28] Bocchio M, Nabavi S, Capogna M (2017). Synaptic plasticity, engrams, and network oscillations in amygdala circuits for storage and retrieval of emotional memories. Neuron..

[29] Frankland PW, Bontempi B, Talton LE, Kaczmarek L, Silva AJ (2004). The involvement of the anterior cingulate cortex in remote contextual fear memory. Science..

[30] Frankland PW, Bontempi B (2005). The organization of recent and remote memories. Nat Rev Neurosci.

[31] Teixeria CM, Pomedli SR, Maei HR, Kee N, Frankland PW (2006). Involvement of the anterior cingulate cortex in the expression of remote spatial memory. J Neurosci.

[32] Matos MR, Visser E, Kramvis I, van der Loo RJ, Gebuis T, Zalm R (2019). Memory strength gates the involvement of a CREB-dependent cortical fear engram in remote memory. Nat Commun.

[33] Lee J, Lee HR, Kim JI, Baek J, Jang EH, Lee J (2000). Transient cAMP elevation during systems consolidation enhances remote contextual fear memory. Neurobiol Learn Mem.

[34] Nakashiba T, Buhl DL, McHugh TJ, Tonegawa S (2009). Hippocampal CA3 output is crucial for ripple-associated reactivation and consolidation of memory. Neuron..

[35] Kitamura T, Ogawa SK, Roy DS, Okuyama T, Morrissey MD, Smith LM (2017). Engrams and circuits crucial for systems consolidation of a memory. Science..

[36] Kwon JT, Jhang J, Kim HS, Lee S, Han JH (2012). Brain region-specific activity patterns after recent or remote memory retrieval of auditory conditioned fear. Learn Mem.

