# Supplementary material for: A critical role of hippocampus for formation of remote cued fear memory
Source: Mol Brain. 2020 Aug 15;13:112. doi: 10.1186/s13041-020-00652-y (PMC7429722; doi:10.1186/s13041-020-00652-y)

Additional Fig. 1 Schematic illustrations of injection cannula placements in mice used for behavior experiments. Related to Fig. 1, 2.

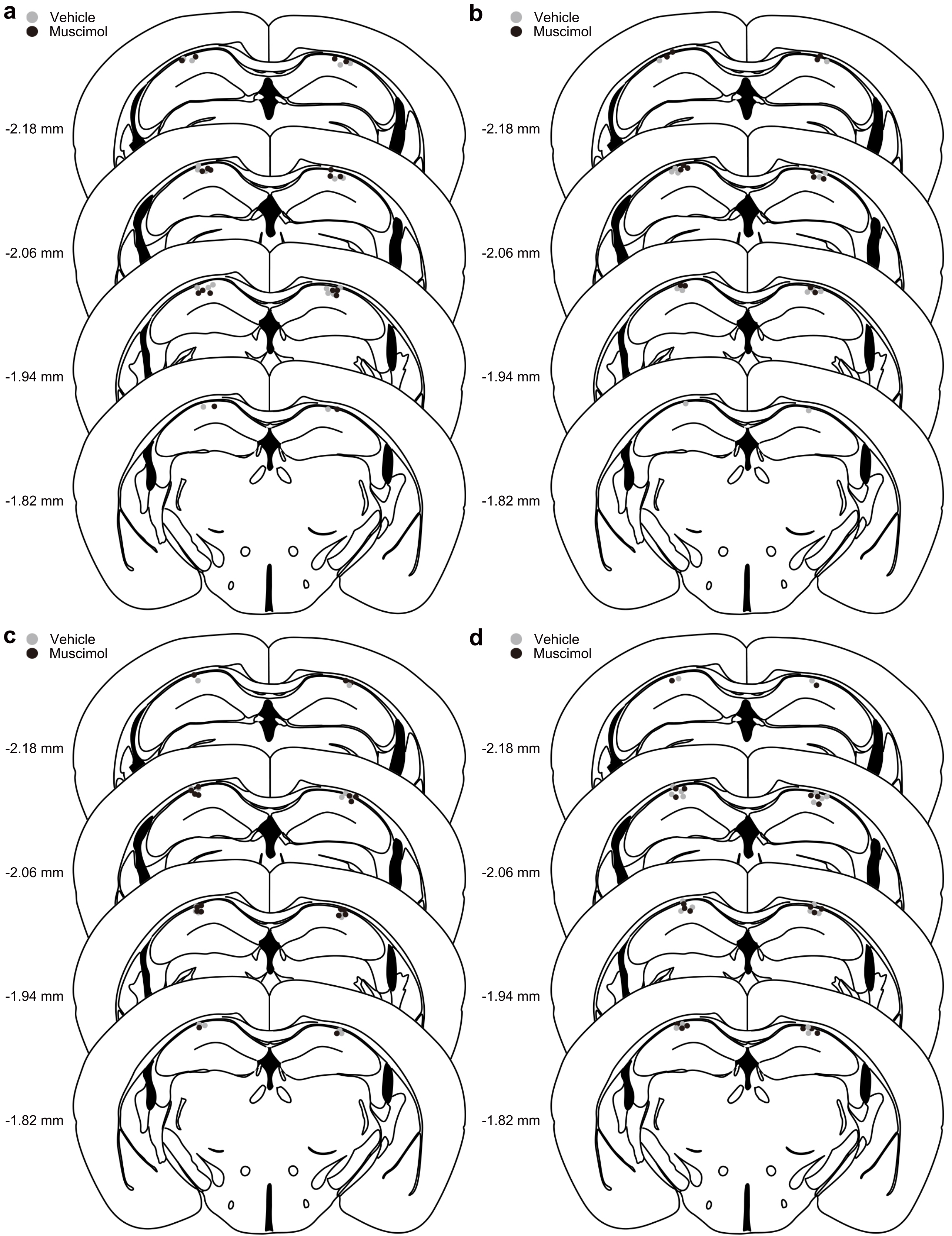

Supplement: Supplementary file 1 — Additional file 1: Figure S1. Schematic illustrations of injection cannula placements in mice used for behavior experiments. Related to Figs. 1, 2. [file 13041_2020_652_MOESM1_ESM.pdf]
