# Supplementary material for: A critical role of hippocampus for formation of remote cued fear memory
Source: Mol Brain. 2020 Aug 15;13:112. doi: 10.1186/s13041-020-00652-y (PMC7429722; doi:10.1186/s13041-020-00652-y)

Additional Fig. 2 Schematic illustrations of injection cannula placements in mice used for behavior experiments. Related to Fig. 3–5.

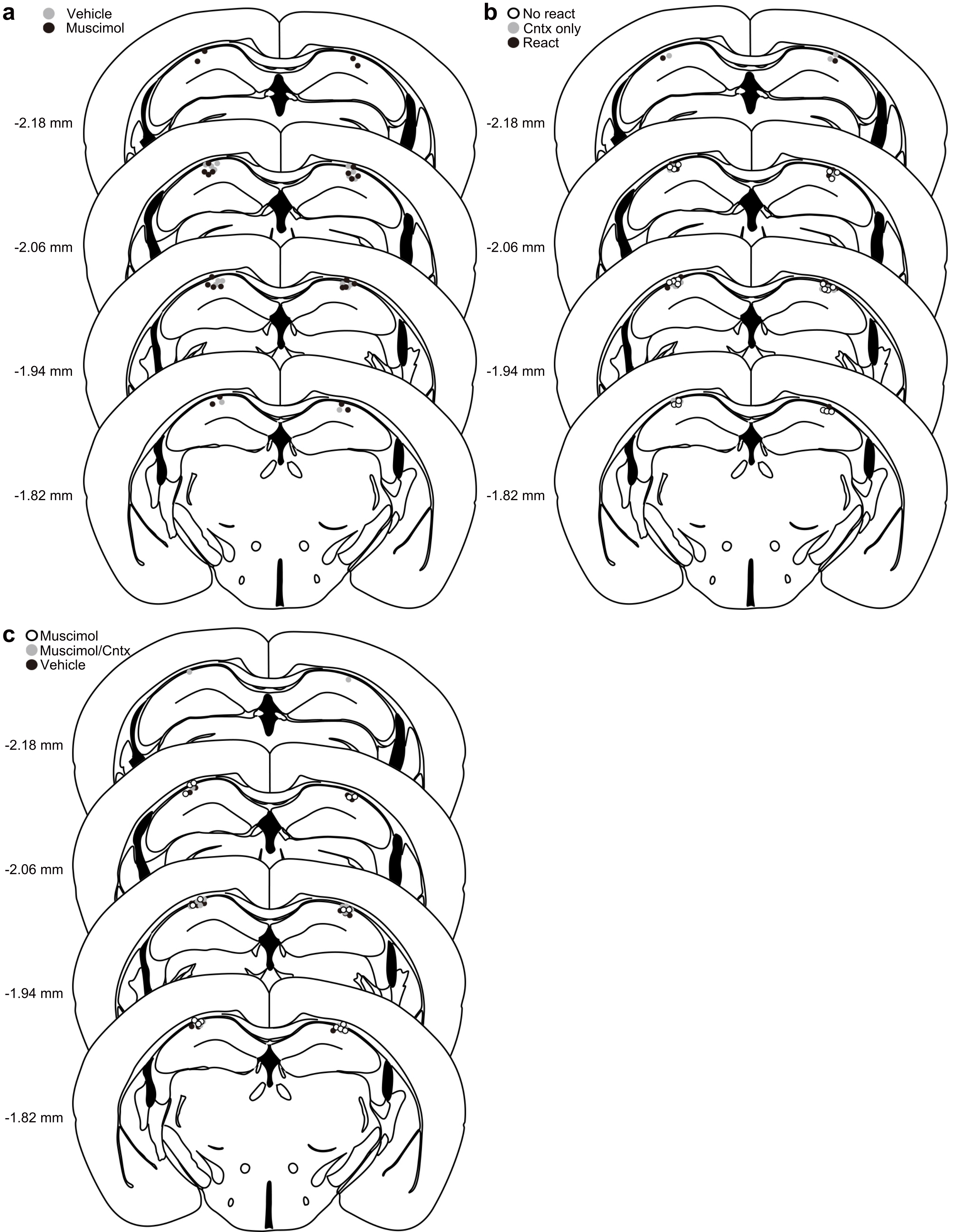

Supplement: Supplementary file 2 — Additional file 2: Figure S2. Schematic illustrations of injection cannula placements in mice used for behavior experiments. Related to Fig. 3, 4 and 5. [file 13041_2020_652_MOESM2_ESM.pdf]
